# Complete mitochondrial genome sequence of the global invasive species *Stictocephala bisonia* (Hemiptera: Membracidae: Smiliinae)

**DOI:** 10.1080/23802359.2021.1911705

**Published:** 2021-05-05

**Authors:** Ruitao Yu, Leining Feng, Xiangqun Yuan

**Affiliations:** Key Laboratory of Plant Protection Resources and Pest Management, Ministry of Education, College of Plant Protection, Northwest A&F University, Yangling, China

**Keywords:** *Stictocephala bisonia*, invasive species, treehopper, mitochondrial genome, phylogenetic analysis

## Abstract

*Stictocephala bisonia* Kopp et Yonke, 1977, an invasive alien species colonizing Taibai County, Shaanxi Province, China, belongs to the subfamily Smiliinae. The total mitogenome sequence size is 15,803 bp in length, consists of 13 protein-coding genes, 22 transfer RNA genes, two ribosomal RNA genes and one control region, and shows a positive AT skew. Phylogenetic analysis results strongly support that treehoppers (Membracidae and Aetalionidae) are monophyletic, and indicate that Smiliinae could be proposed as a separate family.

*Stictocephala bisonia* is native to North America. This Nearctic biological entity appeared in Europe in 1912 for the first time (Horváth 1912), and has gradually become widely distributed since then. This species was initially discovered in Taibai County, Shaanxi Province, China in 2017, which added a newly recorded subfamily to the fauna of China (Yuan et al. 2020). It belongs to the subfamily Smiliinae and the tribe Ceresini. Previous research has focused mostly on the ecology (Krištín 1984, 1987), distribution (Schedl 1991; Lauterer et al. 2011; Walczak et al. 2018), and classification based on morphology (Stegmann 1997). However, the mitogenome sequence of *S. bisonia* has still not been reported and its evolutionary position has not been studied using phylogenetic analysis.

The adult specimens collected in the vicinity of Taibai County (34°05′N, 107°31′E) in July, 2019, were immediately preserved in 100% ethanol and stored in a −80°C freezer before DNA extraction. The voucher specimens were deposited in the Entomological Museum, Northwest A&F University (accession number: Hm-085256; Url: https://ppc.nwafu.edu.cn/english/aboutus/index.htm; Contact person: Xiangqun Yuan, yuanxq@nwsuaf.edu.cn), Yangling, Shaanxi, China. Mitochondrial DNA was sequenced by Illumina HiSeq 4000. In this study, we describe the complete mitogenome sequence of *S. bisonia* based on next-generation sequencing, and our results may contribute to understanding the phylogenetic status of the subfamily Smiliinae within the superfamily Membracoidea. The annotated genomic sequence was submitted to GenBank under accession number MW342606.

The complete assembled mitogenome is a closed circle molecule of 15,803 bp in length, consisting of 37 genes (13 protein-encoding genes (PCGs), 22 transfer RNA (tRNA) genes, two ribosomal RNA (rRNA) genes), and one control region which is 1,586 bp in length. Out of 37 genes, 23 are encoded on the heavy (+) strand while the remainder are on the light (−) strand. For the 13 PCGs, the most common start codon is ATT (*ND1*, *ND2*, *ND3*, *ND4L*, *ND6* and *COX2*), and next is ATG (*ND4*, *COX1*, *COX3*, *ATP6* and *CYTB*). The most common termination codon is TAA (*ND1*, *ND2*, *ND4*, *ND4L*, *ND6*, *ATP6*, *ATP8* and *CYTB*) and next is the incomplete termination codon T (*COX1*, *COX2*, *COX3* and *ND5*). The mitogenome of *S. bisonia* is biased toward a high representation of nucleotides A and T (76.10%), and it shows positive AT and negative GC skews (Supplementary Table S1).

The phylogenetic relationships were estimated using the maximum likelihood method in software PhyloSuite v1.2.2 (Figure 1). It shows that treehoppers (Membracidae and Aetalionidae) are monophyletic with respect to Megophthalminae. Centrotinae and Aetalionidae form a single branch which is sister to Smiliinae, indicating that Smiliinae could be proposed as a separate family, although the evidence is currently insufficient to support this hypothesis, and further studies are required.

**Figure 1.**
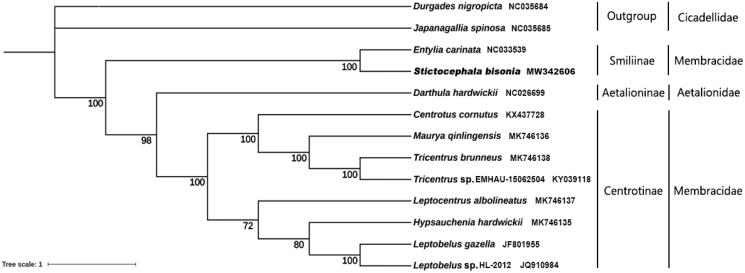
Phylogenetic relationship of *S. bisonia* inferred by ML method based on the concatenated dataset of 13 protein-coding genes.

## Data Availability

The data that support the findings of this study are openly available in GenBank of NCBI at https://www.ncbi.nlm.nih.gov, reference number MW342606. The associated BioProject accession number, SRA data, and BioSample accession number are PRJNA707788, SRR13907305, and SAMN18219192 respectively.
